# A functional sgRNA-CRISPR screening method for generating murine RET and NTRK1 rearranged oncogenes

**DOI:** 10.1242/bio.059994

**Published:** 2023-08-15

**Authors:** Laura Schubert, Anh T. Le, Trista K. Hinz, Andre C. Navarro, Sarah K. Nelson-Taylor, Raphael A. Nemenoff, Lynn E. Heasley, Robert C. Doebele

**Affiliations:** ^1^ Departments of Medicine, University of Colorado Anschutz Medical Campus, Aurora, CO 80045, USA; ^2^ Craniofacial Biology, University of Colorado Anschutz Medical Campus, Aurora, CO 80045, USA; ^3^ Eastern Colorado VA Healthcare System, Rocky Mountain Regional VA Medical Center, Aurora, CO 80045, USA

**Keywords:** CRISPR, Gene fusions, Receptor tyrosine kinase, Tyrosine kinase inhibitor

## Abstract

CRISPR/Cas9 gene editing represents a powerful tool for investigating fusion oncogenes in cancer biology. Successful experiments require that sgRNAs correctly associate with their target sequence and initiate double stranded breaks which are subsequently repaired by endogenous DNA repair systems yielding fusion chromosomes. Simple tests to ensure sgRNAs are functional are not generally available and often require single cell cloning to identify successful CRISPR-editing events. Here, we describe a novel method relying on acquisition of IL3-independence in Ba/F3 cells to identify sgRNA pairs that generate oncogenic gene rearrangements of the *Ret* and *Ntrk1* tyrosine kinases. The rearrangements were confirmed with PCR, RT-PCR and sequencing and Ba/F3 cells harboring *Ret* or *Ntrk1* rearrangements acquired sensitivity to RET and TRK inhibitors, respectively. Adenoviruses encoding Cas9 and sgRNA pairs inducing the *Kif5b-Ret* and *Trim24-Ret* rearrangements were intratracheally instilled into mice and yielded lung adenocarcinomas. A cell line (TR.1) established from a *Trim24-Ret* positive tumor exhibited high *in vitro* sensitivity to the RET inhibitors LOXO-292 and BLU-667 and orthotopic TR.1 cell-derived tumors underwent marked shrinkage upon LOXO-292 treatment. Thus, the method offers an efficient means to validate sgRNAs that successfully target their intended loci for the generation of novel, syngeneic murine oncogene-driven tumor models.

## Teaser

**Summary:** Addressing questions in tumor initiation and experimental therapeutics require immunocompetent systems. A broadly adaptable method for screening sgRNA pairs that induce gene rearrangements encoding murine oncogenic receptor tyrosine kinases is presented.

## INTRODUCTION

Cancer genomes are characterized by numerous and often complex genetic alterations (Hanahan and Weinberg, 2011; Stransky et al., 2014). Dissecting the functional role of each of these alterations can be challenging, but has been crucial to understanding fundamental underpinnings of cancer biology. Small molecule inhibitors are available for treating *ALK*, *ROS1, RET* and *NTRK1-*rearranged receptor tyrosine kinases (RTKs) in lung cancers, but variable or incomplete responses are often observed and acquired resistance is unavoidable (Bivona and Doebele, 2016; Doebele et al., 2015; Drilon et al., 2018; McCoach et al., 2017; Shaw et al., 2013). To this end, human tumor-derived cell lines and patient-derived xenografts (PDXs) have been mainstays for the mechanistic and functional exploration of these genetic alterations as well as drug resistance mechanisms (Davies et al., 2013; Nelson-Taylor et al., 2017; Vaishnavi et al., 2017). The pace of discovery of the oncogenic drivers in histologically-defined cancers such as lung adenocarcinoma is not always matched with access to human cell lines or PDXs as preclinical models. Moreover, a growing literature reveals that the tumor microenvironment (TME) including host immune cells significantly contribute to the therapeutic responses achieved with oncogene-targeted agents like tyrosine kinase inhibitors (TKIs) and KRAS^G12C^-targeted agents (Boumelha et al., 2022; Canon et al., 2019; Kleczko et al., 2023a; Kleczko et al., 2023b; Mugarza et al., 2022; Petroni et al., 2022; Petroni et al., 2021). Thus, rigorous preclinical modeling of oncogene-targeted agents relevant to that observed in patients requires input from the host adaptive immune system. In this regard, experiments with existing human cell lines and PDXs performed in immune-deficient mice present significant limitations that can be overcome with murine models of oncogene-driven cancers permitting direct evaluation of cancer cell-TME interactions in fully immune-competent hosts.

The development of CRISPR/Cas9-based technologies allows precise manipulation of genes in their endogenous loci and is now an essential tool for modeling mutations and chromosomal abnormalities (Sánchez-Rivera and Jacks, 2015). In this system, the Cas9 DNA endonuclease associates with a single guide RNA (sgRNA), thereby directing the complex to a specific locus of the genome via base complementarity. At this locus, Cas9 creates a double stranded break (DSB) upstream of a protospacer adjacent motif (PAM) site (Cho et al., 2013; Cong et al., 2013; Jinek et al., 2012; Jinek et al., 2013; Mali et al., 2013). While CRISPR/Cas9 has been of high impact, it is well documented that off-target effects or inefficient cleavage can occur (Fu et al., 2013). This limitation necessitates rigorous testing of sgRNAs to confirm that they generate their intended alterations. Often, multiple sgRNAs must be tested, which can be both time and labor intensive. Herein, we developed a CRISPR/Cas9 strategy to generate distinct RTK fusion oncogenes that are found across diverse cancer types including lung cancer (Stransky et al., 2014). These murine CRISPR-generated fusion oncogene models as well as others that may be generated in the future are predicted to greatly expand our ability to study mechanisms mediating therapeutic input from the TME and acquired drug resistance in syngeneic, immune-competent mouse models.

## RESULTS

**Fig. 1.**
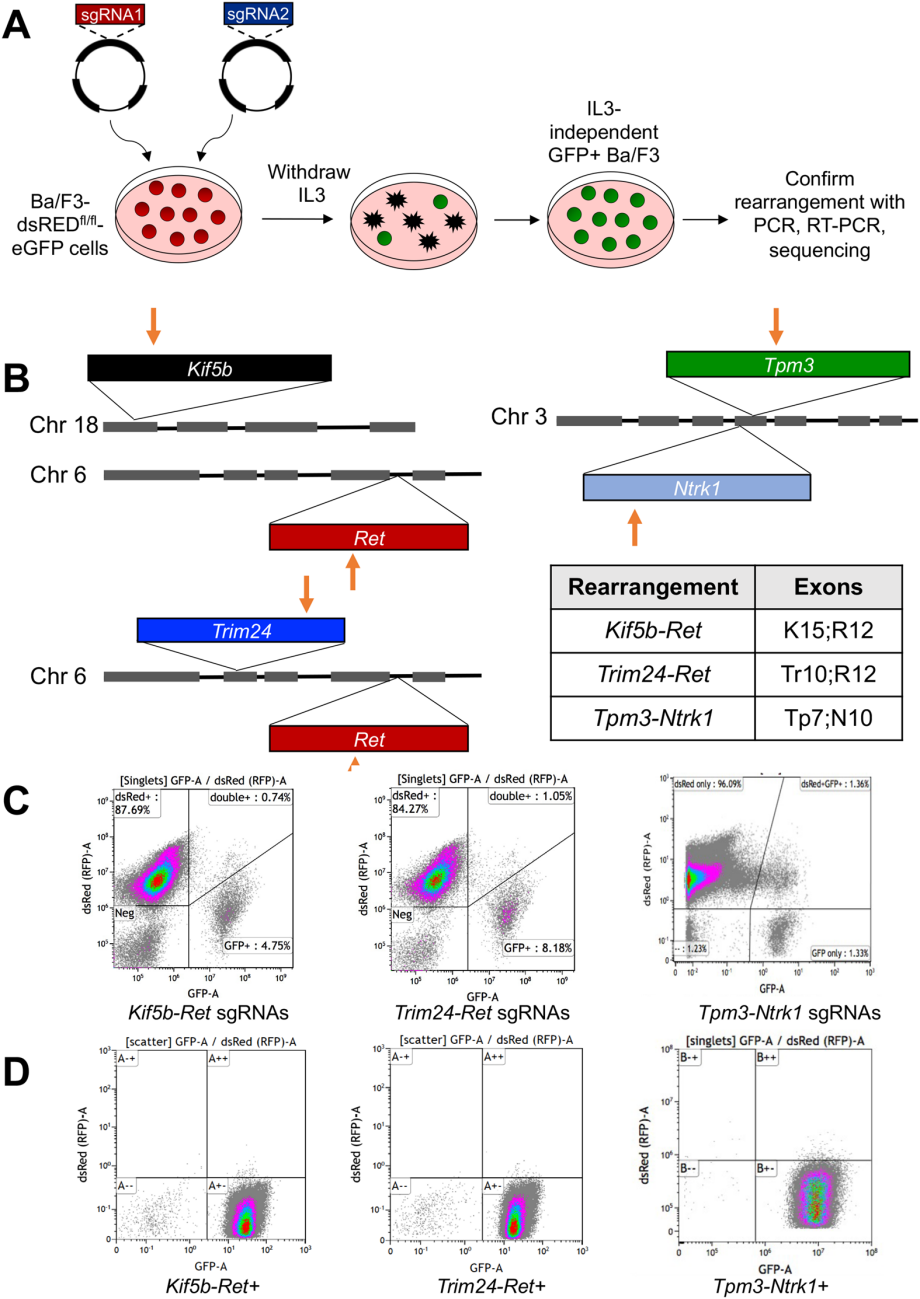
**Ba/F3 based sgRNA screening technique.** (A) Schematic describing sgRNA screening technique in Ba/F3 cells. See text for details. (B) Schematics of CRISPR/Cas9 induced chromosomal rearrangements in *Ret* and *Ntrk1*. Red arrows indicate approximate genomic loci targeted by sgRNAs. Table of specific exons included in *Ret* and *Ntrk* rearrangements (K, *Kif5b*; R, *Ret*; Tr, *Trim24*; Tp, *Tpm3*; N, *Ntrk1*). (C-D) Flow cytometry for dsRED and GFP expression in Ba/F3-dsRED^fl/fl^-eGFP cells 72 h after transfection with sgRNAs (C) and after acquisition of IL3-independence (D). GFP, Green Flourescent Protein; Neg, negative for both GFP and dsRED.

Using CRISPR to engineer chromosomal rearrangements encoding precise fusion oncogenes presents a unique challenge; two independent sgRNAs are necessary to generate double stranded breaks within both the 5′ and 3′ fusion partners. Previous studies have shown that CRISPR systems can be successfully used to generate both inter- and intra-chromosomal rearrangements *in vitro* (Brunet et al., 2009; Choi and Meyerson, 2014; Torres et al., 2014) and *in vivo* using viral delivery methods (Han et al., 2017; Maddalo et al., 2014). Typically, multiple pairs of sgRNAs must be tested to identify those that generate successful fusions. We sought to devise a strategy to quickly validate and enrich for sgRNAs that generate the intended rearrangement. The Ba/F3 murine B-cell line is dependent on exogenous IL3 for growth and proliferation, but can be rendered IL3-independent upon expression of oncogene drivers. We hypothesized that IL3-independence in Ba/F3 cells could be used to screen and select for sgRNAs that successfully generate oncogenic alterations. Briefly, Ba/F3 cells are co-transfected with a pair of sgRNA-encoding plasmids targeting the 5′ and 3′ fusion partners, IL3 is removed, and cells are cultured until IL3-independent clones emerge (Fig. 1A). Natural selective pressures enrich for productive sgRNAs pairs that yield an oncogenic RTK fusion, thereby eliminating the need for single cell cloning to validate sgRNAs. Because Ba/F3 cells are a murine cell line, they are ideal for testing sgRNAs targeting the mouse genome that can subsequently be used for downstream *in vivo* applications. This system also allows for rapid and efficient testing of different permutations of sgRNA pairs with limited cloning steps, which is especially useful when generating fusions.

To monitor transfection efficiency, we transduced Ba/F3 cells with pMSCV-loxp-dsRED-loxp-eGFP-Puro-WPRE, which encodes a floxed dsRED gene followed by an eGFP gene (Koo et al., 2011). After transduction with the conditional eGFP expression virus, we selected dsRED+ cells using FACS (Fig. S1). This results in a stable cell line, Ba/F3-dsRED^fl/fl^-eGFP, labeled with dsRED until Cre recombinase is expressed, which excises the dsRED gene and allows transcription of eGFP yielding dsRED-, eGFP+ cells. Finally, to use eGFP positivity as a surrogate for successful transfection, we engineered the pX330 Cas9-containing plasmid to also contain the Cre recombinase gene. As a positive control for the method, we tested validated sgRNAs described by Maddalo et al. that generate *Eml4-Alk* rearrangements both *in vitro* and *in vivo* (Maddalo et al., 2014). We transfected Ba/F3-dsRED^fl/fl^-eGFP cells with the pX330-*Alk-Eml4* plasmid containing both *Eml4* and *Alk* sgRNAs. Flow cytometry was used to assess transfection efficiency and presence of GFP+ cells (Fig. S2A). After approximately 3 weeks, IL3-independent cells were proliferating and validated for the *Eml4-Alk* rearrangement with genomic PCR (Fig. S2B). Ba/F3+*Eml4-Alk* cells were ∼eight times more sensitive to the ALK inhibitor crizotinib, than the parental Ba/F3-dsRED^fl/fl^-eGFP cell line supplemented with IL3 (Fig. S2C). Additionally, the resulting *Eml4-Alk*+ population was almost entirely GFP+ (Fig. S2D). These findings confirm that the sgRNA screening method successfully selects for cells that have acquired an oncogenic rearrangement.

**Fig. 2.**
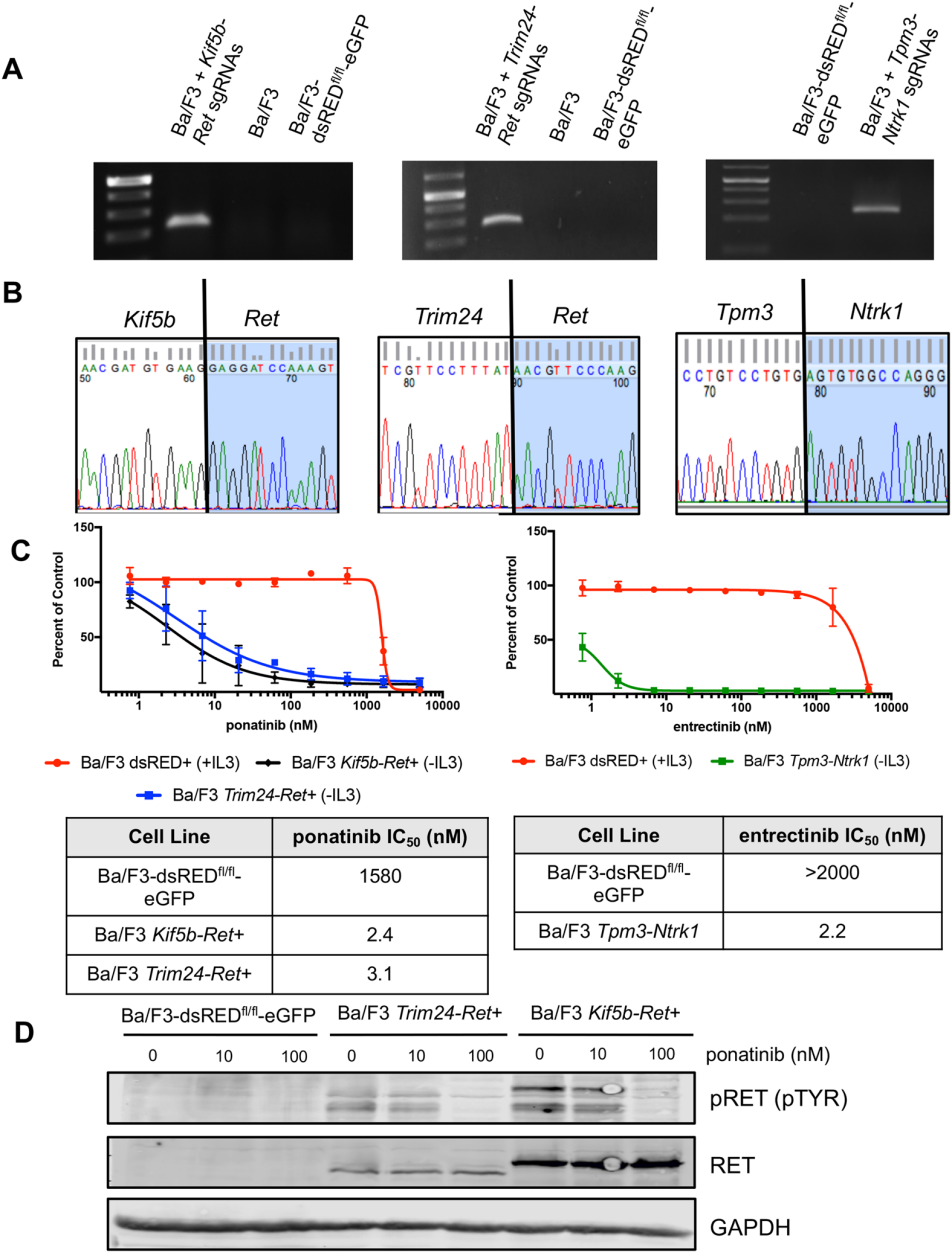
**Ba/F3 cell-based screening identifies pairs of sgRNAs that successfully generate TKI-sensitive *Kif5b-Ret*, *Trim24-Ret* and *Tpm3-Ntrk1* rearrangements.** (A) Fusion specific RT-PCR for *Kif5b-Ret,* genomic PCR for *Trim24-Ret* or *Tpm3-Ntrk1*. (B) Sequencing across fusion point in cDNA from Ba/F3 *Kif5b-Ret*+ cells and genomic DNA from *Trim24-Ret* and *Tpm3-Ntrk1.* (C) (right) MTS proliferation assay performed on Ba/F3 dsRED+, Ba/F3 *Kif5b-Ret*+ and Ba/F3 *Trim24-Ret*+ cell treated with increasing doses of ponatinib. *N*=3 error bars represent ±s.e.m. (left) MTS proliferation assay performed on Ba/F3 dsRED+ and Ba/F3 *Tpm3-Ntrk1*+ cells treated with increasing doses of entrectinib. *N*=3 biological replicates with three technical replicates each, error bars represent ±s.e.m. The IC_50_ values are tabulated beneath the dose-response curves and were calculated with the Prism software program. (D) Immunoblot analysis of Ba/F3 dsRED+, *Trim24-Ret*+ and Ba/F3 *Kif5b-Ret+* cells treated with ponatinib for 2 h. Within the figure, Ba/F3 dsRED+ indicates Ba/F3-dsRED^fl/fl^-eGFP cells.

A central goal of this study was to generate novel murine *Ret* and *Ntrk1* cancer models. These oncogenes are observed in NSCLC and other cancer types, but no murine models and limited *RET*+ and *NTRK*+ human cell lines exist (Drilon et al., 2018; Kohno et al., 2012; Lipson et al., 2012; Schubert et al., 2021; Takeuchi et al., 2012; Vaishnavi et al., 2013). We designed sgRNAs predicted to generate the murine equivalents of three distinct fusion oncogenes, *Kif5b-Ret*, *Trim24-Ret* and *Tpm3-Ntrk1* (Fig. 1B). We established that the Ba/F3-dsRED^fl/fl^-eGFP cells were successfully transfected with several combinations of sgRNA pairs, as assessed by flow cytometry for GFP+ cells (Fig. 1C). In each case, we were successful in generating IL3-independent Ba/F3 cells in approximately 4-5 weeks which were nearly 100% GFP+ (Fig. 1D). We believe that IL3-independent cells took longer to establish relative to the *Eml4-Alk* cells because the transfected sgRNA pairs were encoded on separate plasmids, while the *Eml4-Alk* sgRNAs were both encoded within a single plasmid. We confirmed that IL3-independent Ba/F3 cells harbored their intended chromosomal rearrangements with fusion-specific genomic PCR or RT-PCR (Fig. 2A). Targeted sequencing of genomic DNA or cDNA further validated that *Kif5b-Ret*, *Trim24-Ret* and *Tpm3-Ntrk1* fusions were present (Fig. 2B). It is interesting to note that with both *Ret* fusions, one copy of wild-type, non-rearranged *Ret* was preserved in the Ba/F3 cells (Fig. S3).

We demonstrated that *Kif5b-Ret*+ and *Trim24-Ret*+ Ba/F3 cells were sensitive to multiple RET inhibitors, including ponatinib, cabozantinib, alectinib and foretinib (Fig. 2C, Fig. S4A-D). We were also able to detect the expression of the RET fusion protein in *Trim24-Ret*+ and *Kif5b-Ret*+ Ba/F3 cells (Fig. 2D). As expected, phosphorylation of RET could be inhibited upon treatment with ponatinib, a pan-TKI with RET activity (Fig. 2D). Similarly, *Tpm3-Ntrk1*+ Ba/F3 cells were significantly more sensitive to the TRK inhibitor entrectinib than parental Ba/F3 sdRED+ cells (Fig. 2C). Collectively, these data demonstrate that this method can efficiently screen and enrich for pairs of sgRNAs that generate distinct and functional murine equivalents of known human fusion oncogenes.

**Fig. 3.**
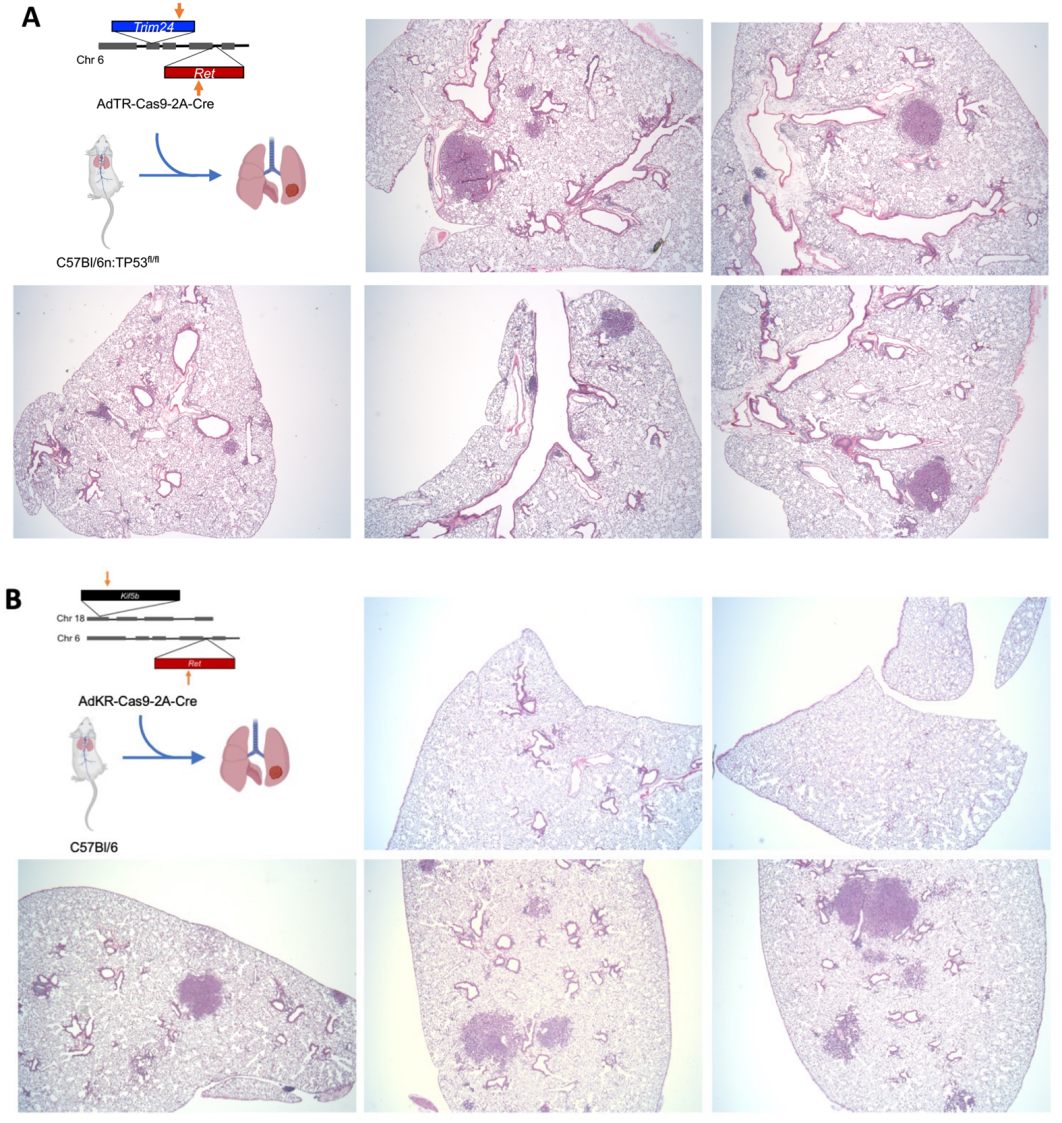
**Murine models of *Trim24-Ret* and *Kif5b-Ret* fusion lung cancer.** Design schema of CRISPR/Cas9 strategy to induce rearrangement at the (A) *Trim24* and *Ret* gene locus and (B) *Kif5b* and *Ret* for the generation of *Trim24-Ret* or *Kif5b-Ret* fusion respectively. (A) Trp53^fl/fl^ mice were intratracheally instilled with AdTR-Cas9-2A-Cre adenovirus and after 6 weeks, lung lobes were harvested, formalin-fixed, paraffin-embedded and sections were stained with H&E to assess histology. (B) C57BL/6 mice (wild-type Trp53) were intratracheally instilled with Ad-KR-Cas9-2A-Cre adenovirus and after 10 weeks lung lobes were harvested, formalin-fix, paraffin-embedded and sections were stained with H&E.

To test the ability of the validated pairs of *Trim24* or *Kif5b*-*Ret* sgRNAs to induce lung tumors in mice, the sgRNA pairs shown in Table S1 were cloned into the Ad-Cas9-2A-Cre adenoviral vector encoding Cas9 to catalyze the intended CRISPR-dependent gene rearrangements and Cre recombinase to induce LoxP-mediated excision of floxed alleles. Six weeks after instillation of Adeno-Cas9-Cre-*Trim24/Ret* gRNA virus in C57BL/6-TP53^fl/fl^ mice, multiple tumor foci were observed in the fixed lung lobes (Fig. 3A), demonstrating that the *Trim24* and *Ret* sgRNAs induced gene rearrangement-dependent tumorigenesis as previously observed with *Eml4-Alk* rearrangements (Kleczko et al., 2023a; Maddalo et al., 2014). Hematoxylin and Eosin (H&E) staining confirmed the presence of foci with dense, proliferating cells with a loss of normal lung tissue architecture, consistent with neoplastic growth. Similarly, we introduced an adenovirus expressing the *Kif5b* and *Ret* sgRNAs into lungs of C57BL/6 mice without floxed *Trp53* and observed multiple tumors 10 weeks after instillation of adenovirus (Fig. 3B). Thus, the *Trim24-Ret* and *Kif5b-Ret* rearrangements efficiently induce lung tumors in mice.

**Fig. 4.**
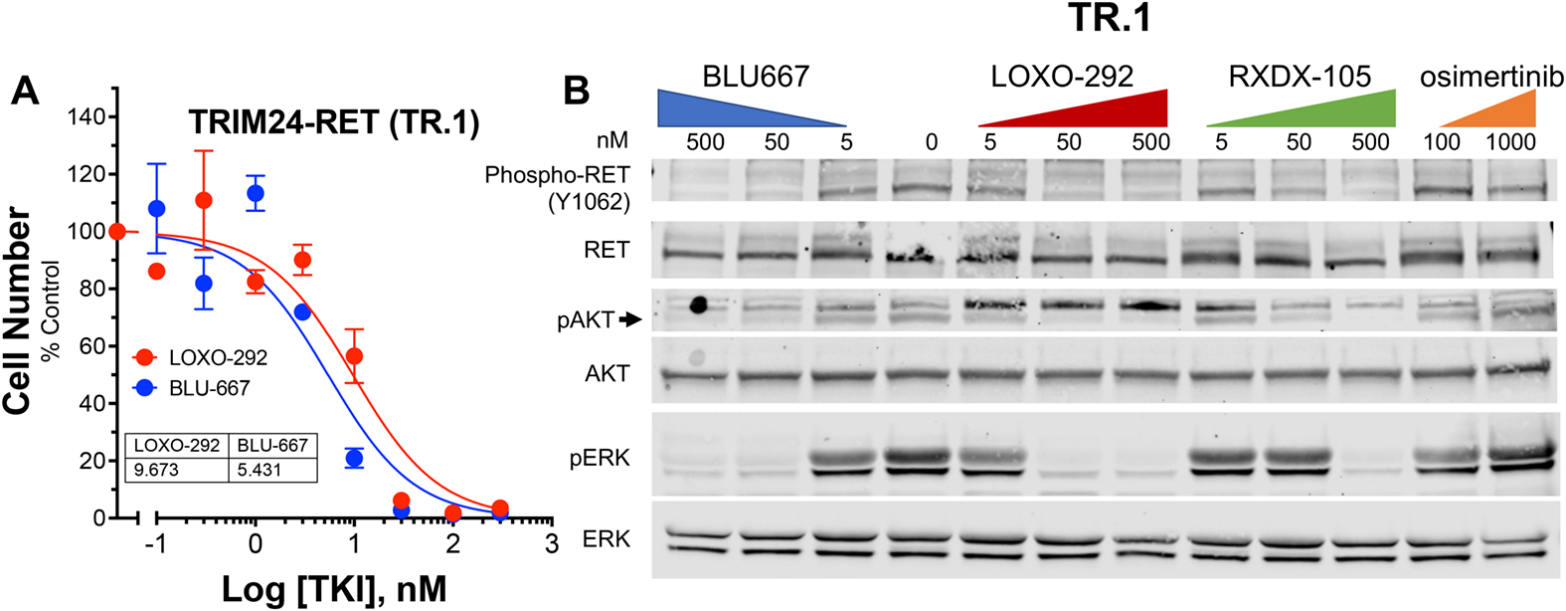
**Analysis of novel murine *Trim24-Ret* fusion cell line.** (A) TR.1 cells were seeded at 200 cells/well in 96-well plates and after 24 h, treated for 7 days with LOXO-292 or BLU-667 at the indicated concentrations. Cell number was determined with CyQUANT reagent as described in the Materials and Methods. The data are the means and SEM of three biological replicates, each with three technical replicates. (B) TR.1 cells were treated for 2 h with the indicated TKIs and cell extracts were submitted to immunoblot analysis for phospho-Y1062-RET, phospho-Ser473 AKT and phospho-ERK1/2 as described in the Materials and Methods. The filters were subsequently stripped and reprobed for total RET, AKT and ERK.

**Fig. 5.**
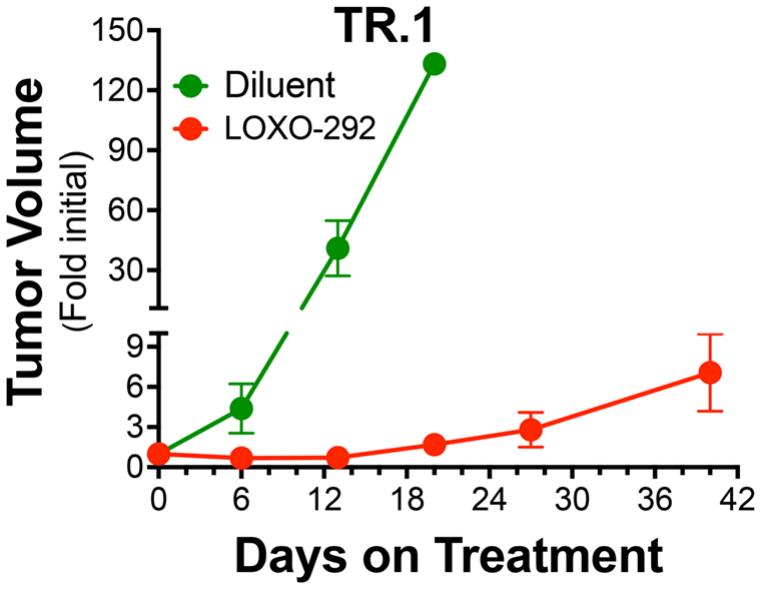
**Sensitivity of TR.1 cell-derived orthotopic tumors to LOXO-292.** TR.1 cells (500,000 cells per mouse) were implanted into the left lungs of C57BL/6 mice. The orthotopic tumors were allowed to establish for ∼10 days and the mice were submitted to μCT imaging to determine pre-treatment tumor volumes. Following randomization (*n*=5 per group), the mice were treated daily with 10 mg/kg LOXO-292 or diluent by oral gavage. Mice were imaged by μCT weekly over the course of the experiments and tumor volume is presented as the fold change from the initial pre-treatment measurement. The initial tumor volumes (mean±s.e.m.) for the diluent and LOXO-292-treated groups were 2.4±0.7 and 4.3±1.2 mm^3^, respectively. The necessity for euthanasia of multiple mice in the diluent-treated group by day 13 prevented a full two-way ANOVA of the dataset. However, Mann–Whitney analysis of diluent and LOXO-292 treatment at day 6 revealed a significant difference (*P*=0.0079). Also, a Kruskal–Wallis test of the LOXO-292-treated mice over time revealed that tumor volumes on days 6 and 13 were different from day 40 (*P*=0.0094 and 0.0082, respectively), demonstrating statistically significant progression on continuous treatment.

To develop a murine *Trim24-Ret*-driven lung adenocarcinoma cell line, tumor-bearing lungs were harvested, minced and submitted to standard tissue culture techniques. A stable cell line was subsequently established, TR.1, that exhibited potent *in vitro* growth inhibition (Fig. 4A) in response to the RET-specific TKIs (Subbiah et al., 2020), LOXO-292 (selpercatinib) and BLU-667 (pralsetinib). Immunoblot analyses of extracts from TR.1 cells treated for 2 h with *RET*-active TKIs (BLU-667, LOXO-292 and RXDX-105) and the third-generation EGFR inhibitor, osimertinib, as a negative control demonstrated that the three *RET* inhibitors decreased levels of phospho-Y1062 RET, phospho-S473-AKT and phospho-ERK in a dose-dependent fashion, while osimertinib was without effect (Fig. 4B). The TR.1 cell line was used to establish orthotopic tumors in the left lung lobe of syngeneic C57BL/6 mice (see Materials and Methods and Kleczko et al., 2023a and b). Following tumor establishment for ∼10 days, the initial tumor volumes were measured by μCT and the mice were treated with LOXO-292 (10 mg/kg) or diluent by oral gavage. Treatment for 6 and 13 days with LOXO-292 induced tumor shrinkage to 31.4±9.1% and 27.4±5.2% of control, respectively (Fig. 5). Within three weeks of LOXO-292 treatment, the TR.1 tumors began to progress, albeit at a slower rate than that exhibited by untreated tumors. The studies demonstrate that the TR.1 cell line is suitable for pursuing a variety of pre-clinical studies using an orthotopic implantation model.

## DISCUSSION

The Ba/F3 cell-based sgRNA screening method presented here allows for the validation of sgRNAs prior to dedicating time and resources to larger experiments or *in vivo* studies. We believe that this technique will be extremely versatile and that it can be used to select for other types of oncogenic alterations, such as point mutations or small insertions/deletions. For example, the design of sgRNA pairs that result in deletion of MET exon 14 to yield the “MET exon 14 skip” oncogene should be straightforward and yield murine models for this targetable oncogene. The generation of murine cell lines driven by *Kif5b-Ret* and *Tpm3-Ntrk1* fusion oncogenes is presently ongoing. This method poses some potential limitations, including the inability to screen interesting non-transforming alterations, and the possibility that Ba/F3 cell chromatin structure may limit accessibility to certain genomic loci. We attempted but failed to identify pairs of sgRNAs to induce distinct *Ros1* fusion oncogenes (including *Gopc-Ros1, Sdc4-Ros1* and *Ezr-Ros1*) with this method and are developing Cre-inducible *Cd74-Ros1* and *Ezr-Ros1*-transgenic mice as an alternative strategy. We suspect that other genetic alterations and/or protein expression profiles that are not present in Ba/F3 cells may be required for some transforming events. Indeed, a recent study demonstrated that certain *ERBB* gene fusions could not transform Ba/F3 cells but were oncogenic in human cell models (Schubert et al., 2023). Overall, this strategy provides a simple and efficient protocol for validating oncogenic sgRNAs that should accelerate functional characterization of many transforming alterations. Ultimately, the application of this CRISPR/Cas9 strategy will permit the development of mice and murine lung cancer cell lines driven by relevant oncogenes described in patients for which human cell line and PDX equivalents are not readily available.

We have recently reported the development of murine *Eml4-Alk*-driven lung cancer cell lines that readily form orthotopic lung tumors in C57BL/6 mice (Kleczko et al., 2023a). Notably, the orthotopically-implanted tumors in syngeneic hosts exhibited profound and durable shrinkage upon alectinib therapy, but transient responses in immune-deficient mice, thereby demonstrating a role of adaptive immunity in the therapeutic response to TKIs. A similar requirement for adaptive immunity is observed with murine lung cancer cell lines driven by oncogenic *Egfr* transgenes (Kleczko et al., 2023b). In fact, there is a growing literature supporting the requirement for host immunity in the therapeutic responses of cancers to diverse oncogene-targeted agents (Petroni et al., 2022; Petroni et al., 2021). Thus, development and deployment of transplantable murine equivalents of oncogene-driven human cancers will permit a mechanistic investigation of these observations in fully immune-competent mice where similar studies using human cancer cell lines or PDXs will require immune-deficient mouse strains reconstituted with humanized immune systems.

Compared to the durable and profound TKI responses observed in mice bearing orthotopic *Eml4-Alk* and *Egfr^del19^* tumors (Kleczko et al., 2023 a and b), the depth of response of the *Trim24-Ret* cell line, TR.1, to LOXO-292 described herein was quite modest (Fig. 5). Moreover, clear evidence of tumor progression after ∼2 weeks of LOXO-292 therapy was observed, suggesting rapid acquisition of TKI resistance. In a recent study with murine *Egfr^del19^* cell lines, we explored mechanisms that may mediate rapid progression of the tumors under continuous osimertinib treatment when propagated in immune-deficient mice (Kleczko et al., 2023b). Induction of HGF and MET expression was observed in the progressing tumors and a cell line established from a TKI-resistant tumor exhibited acquired sensitivity to the MET inhibitor, crizotinib. As MET pathway activation is considered a bona fide bypass resistance pathway in human EGFR-driven lung cancers, murine oncogene-driven lung tumors propagated in immune-competent hosts may represent tractable model systems to explore acquired drug resistance and to define novel combination therapy strategies for prolonging progression free survival.

This approach also allows for efficient, simultaneous manipulation of other mutations, such as loss of tumor suppressors. There is a growing understanding that the genetic context in which driver oncogenes occur can substantially alter the tumor biology. The role of tumor suppressor loss in relationship to therapy response, development of resistance and interaction with the tumor microenvironment is in need of further investigation. In particular, it has been shown that tumor suppressors have differential ability to promote oncogenesis in the context of *KRAS* mutated lung cancer with distinct therapeutic vulnerabilities (Cai et al., 2021; Yousefi et al., 2022). We found that our *Trim24-Ret* rearrangement, which was introduced in mice bearing floxed *Tp53* genes, formed tumors much more rapidly than our *Kif5b-Ret* rearrangements in *Tp53* wild-type mice (6 weeks versus 10 weeks). Indeed, this CRISPR-induced fusion method technique could be paired with novel technologies such as that employed by Cai et al. (2021) to explore the functional impact of tumor suppressors on oncogenic RTKs without the need to generate genetically engineered mouse models for each oncogene.

## MATERIALS AND METHODS

### Ba/F3 cell culture and transfection

As previously described (Vaishnavi et al., 2013), Ba/F3 cells were cultured in RPMI1640 (Corning) supplemented with 10% fetal bovine serum (FBS) and 1 ng/ml IL3 (R&D Systems) where indicated. Cells were maintained in a humidified incubator at 37°C and 5% CO_2_. Parental Ba/F3 cells were authenticated by routinely assessing IL3-dependence for cell growth, and tested for mycoplasma every 3-4 months using the MycoAlert assay (Lonza).

The pMSCV-loxp-dsRED-loxp-eGFP-Puro-WPRE construct (a gift from Hans Clevers, Oncode Institute, Hubrecht Institute, Royal Dutch Academy of Science and University Medical Center Utrecht, The Netherlands; Addgene plasmid #32702) was stably introduced into Ba/F3 cells via retroviral transduction followed by puromycin selection. The resulting transduced cell line, referred to as Ba/F3-dsRED^fl/fl^-eGFP in this study, is available upon request. Fluorescence-activated cell (FAC)-sorting of single cell suspensions was used to select for dsRED+ cells after transduction. Cells were suspended in PBS with 1 mM EDTA, 25 mM HEPES (pH 7.0) and 1% FBS and sorted with the Astrios EQ (Beckman Coulter) into RPMI1640 (Corning) with 10% FBS at the CU Cancer Center Flow Cytometry Shared Resource. Ba/F3-dsRED^fl/fl^-eGFP cells were then transfected with 5 µg of total sgRNA plasmid using the Mirus Ingenio Electroporation Kit (described below) with the BioRad GenePulser Xcell set at 300 V and 950µF. Three days post-transfection, cells were suspended in PBS and analyzed for GFP and dsRED expression by flow cytometry on the YETI (Propel Labs) at the CU Cancer Center Flow Cytometry Shared Resource and analyzed using Kaluza Software (Beckman Coulter). Gating was performed to capture live cells, then analyzed for dsRED and GFP positivity. One hundred thousand events were captured per cell population.

### Instillation of recombinant adenoviruses into murine lungs and establishment of the TR.1 murine *Trim24-Ret* lung cancer cell line

A recombinant adenovirus construct (pAV-U6-Trim24-U6-Ret,CMV-hCas9:P2A:Cre; VectorBuilder, Chicago, IL, USA) encoding Cas9, Cre recombinase and the *Trim24-Ret* and Kif5b-Ret sgRNA pairs shown in  Table S1 were packaged by ViraQuest, Inc (North Liberty, IA, USA). C57BL/6 mice bearing wild-type or floxed TP53 alleles (B6.129P2-Trp53^tm1Brn^/J; Jackson Laboratory stock #008462) were submitted to intratracheal instillation using IACUC-approved methods as described (DuPage et al., 2009) with 50 μl Adeno-TR-Cas9-Cre and Adeno-KR-Cas9-Cre viruses at a dose of 1.5×10^8^ PFU/mouse. Lungs from tumor-bearing mice 6 weeks after Adeno-TR-Cas9-Cre-infection were harvested and tumors minced, digested and cultured in RPMI-1640 medium containing 5% fetal bovine serum until a stable epithelial cell line (TR.1) was established. The presence of the predicted *Trim24-Ret* fusion protein was confirmed by anti-Ret immunoblot analysis. The murine TR.1 cell line is available upon request.

### Inhibitors and reagents

Ponatinib, cabozantinib and foretinib were purchased from SelleckChem (Houston, TX, USA). Alectinib was obtained from Chugai Pharmaceuticals and entrectenib was obtained from Igynta. LOXO-292 and BLU-667 were purchased from MedChemExpress (Monmouth Junction, NJ, USA). The RET antibody (EPR2871) was purchased from Abcam, pRET Y1062 (sc-20252) was purchased from Sana Cruz Biotechnology (Dallas, TX, USA) and GAPDH (65C) were purchased from Millipore.

### sgRNA design and cloning

The pX330-U6-Chimeric_BB-CBh-hSpCas9 plasmid (a gift from Feng Zhang; Addgene plasmid #42230) was modified to express a bicistronic peptide containing Cas9 and Cre as follows. pX330 was sequentially modified to accept a 2.4 kb Cas9-P2A-Cre fragment from pSECC (a gift from Tyler Jacks; Addgene plasmid #60820) first by an EcoRI (New England Biolabs) digestion with a subsequent fill-in reaction by DNA polymerase and then a AccIII restriction digest. The 2.4 kb insertion fragment was generated from pSECC by a restriction digest with AccIII (Promega) and SacII (New England Biolabs). Ligation of this fragment into the pX330 modified plasmid was performed using T4 DNA Ligase (Invitrogen) in an overnight reaction at 16°C. The ligated product was then used to transform competent bacteria. sgRNAs targeting *Trim24, Kif5b, Tpm3, Ret* and *Ntrk1* were designed using the Zhang lab CRISPR design tool (crispr.mit.edu) and are presented in Table S1. The pX330+Cre plasmid was digested with BbsI (New England BioLabs) and ligated to annealed and phosphorylated sgRNA oligonucleotides (Integrated DNA Technologies). Ligated plasmids were transformed into DH5α *E.coli* (Life Technologies). The PX330-*Alk-Eml4* plasmid was kindly provided by Andrea Ventura (Maddalo et al., 2014).

### Proliferation assays

Ba/F3-dsRED^fl/fl^-eGFP cells that were transfected with sgRNA plasmids and selected for IL3-independence were plated (10,000 cells per well) in 96-well tissue culture plates in RPMI1640 (Invitrogen) supplemented with 10% FBS, with or without 1 ng/ml IL3 where indicated and treated with the indicated concentrations of drugs for 72 h. CellTiter 96 MTS was used to estimate cell numbers according to the manufacturer's instructions (Promega). Each assay was performed in triplicate with three biological replicates. For analysis of the sensitivity of the murine *Trim24-Ret* lung cancer cell line, TR.1, cells were seeded (200 per well) in 96-well plates and 24 h later, incubated for 7 days in triplicate with increasing doses (0-300 nM) of LOXO-292 or BLU-665. Cell number was assessed by measuring DNA content using the CyQUANT Direct Cell Proliferation Assay (Life Technologies, #C35011, Carlsbad, CA, USA) according to manufacturer's instructions. IC_50_ values were calculated using GraphPad Prism software.

### Immunoblotting

Immunoblotting was performed as previously described (Davies et al., 2013). Briefly, cells were lysed in radioimmunoprecipitation assay buffer (RIPA) supplemented with Halt protease and phosphatase inhibitor (Thermo Scientific). Lysates were diluted in 4X protein sample loading buffer (LI-COR) and subjected to SDS-PAGE. Proteins were transferred to nitrocellulose and stained with primary antibodies followed by IRDye anti-mouse or anti-rabbit IgG (LI-COR). The Odyssey Imager and Odyssey Image Studio Software (LI-COR) were used to scan membranes and analyze images.

### DNA isolation, PCR and sequencing

DNA was isolated using the Quick-gDNA MiniPrep Kit from Zymo Research according to the manufacturer's instructions. PCR was performed with primers designed to detect *Eml4-Alk, Eml4, Trim24-Ret, Tpm3-Ntrk1* and *Ret.* PCR products were separated with electrophoresis on a 1% agarose gel. Standard Sanger sequencing was performed on PCR-amplified fragments.

### RNA isolation, RT-PCR and sequencing

RNA was isolated using the RNEasy plus MiniKit (Qiagen) according to the manufacturer's instructions. cDNA was generated using the SuperScript III First-Strand Synthesis System (Invitrogen) using random hexamers as per the manufacturer's instructions. PCR reactions were performed with primers designed to detect *Kifb5-Ret* fusion transcripts. PCR products were separated with electrophoresis on a 1% agarose gel. PCR amplified-cDNA was subjected to standard Sanger sequencing.

### Orthotopic mouse model of RET lung cancer

The TR.1 cell line was propagated as orthotopic tumors in the left lung lobe of 9-week-old female C57BL/6 mice (WT; C57BL/6J; #000664, Jackson Laboratory, Bar Harbor, ME, USA) using IACUC-approved protocols. Cells were prepared in a solution of 1.35 mg/ml Matrigel (Corning #354234) diluted in Dulbecco's PBS (Corning) for injection. Mice were anesthetized with isoflurane, the left side of the mouse was shaved, and a 1mm incision was made to visualize the ribs and left lobe of the lung. Using a 30-gauge needle, 2.5×10^5^ cells were injected in 40µL of Matrigel-cell mixture directly into the left lobe of the lung and the incision was closed with staples. Tumors were permitted to establish for 10 days and then the mice were submitted to micro-computed tomography (µCT) imaging to obtain pre-treatment tumor volumes. Tumor-bearing mice were randomized into treatment groups (*n*=5), either 10mg/kg selpercatinib/LOXO-292 or diluent control (0.5% HPMC) by oral gavage 5 days per week until the end of study. Mice were imaged weekly by µCT imaging to monitor effects of drug treatment on tumor volume. Tumor volume µCT imaging was performed by the Small-Animal IGRT Core at the University of Colorado Anschutz Medical Campus in Aurora, CO, USA using the Precision X-Ray X-Rad 225Cx Micro IGRT and SmART Systems (Precision X-Ray, Madison, CT, USA). Tumor volume was quantified from µCT images using ITK-SNAP software^36^ (www.itksnap.org). Upon study end mice were sacrificed using CO_2_ and cervical dislocation as a secondary method.

## Supplementary Material

10.1242/biolopen.059994_sup1 Supplementary information
