# Supplementary material for: A functional sgRNA-CRISPR screening method for generating murine RET and NTRK1 rearranged oncogenes
Source: Biol Open. 2023 Aug 15;12(8):bio059994. doi: 10.1242/bio.059994 (PMC10445739; doi:10.1242/bio.059994)
Supplement: Supplementary information [file biolopen-12-059994-s1.pdf]

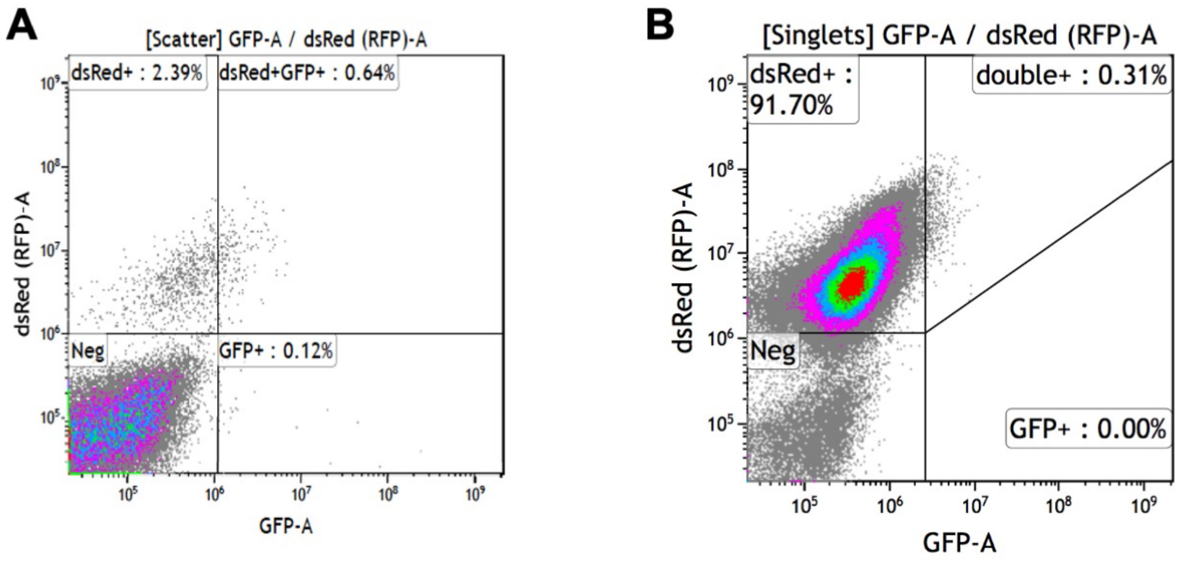

Supplementary Figure 1

Fig. S1. Transduction and selection for Ba/F3-dsRED$^{fl/fl}$-eGFP cells. A. Flow cytometry analysis for dsRED+ and eGFP+ Ba/F3 cells three days post transduction with pMSCV-loxp-dsRED-loxp-eGFP-Puro-WPRE. **B.** dsRED+ Ba/F3 cell population after FACS selection for dsRED+ cells.

Biology Open • Supplementary information

A

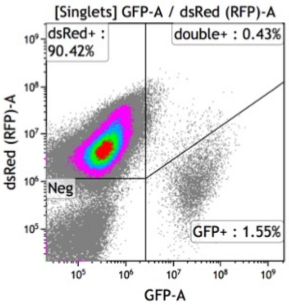

72 hours post-transfection with *Eml4-Alk* sgRNAs

B

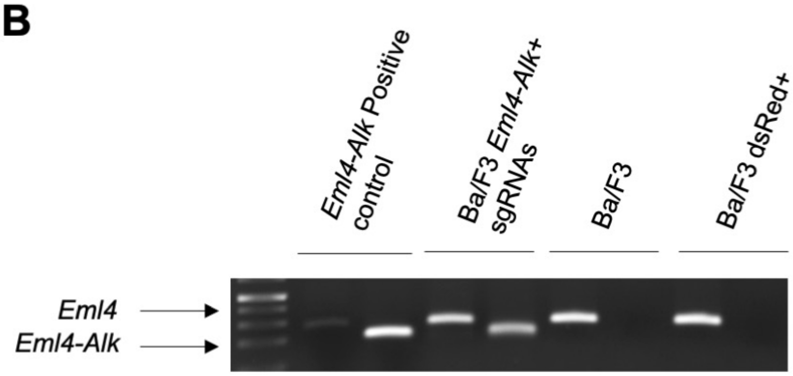

C

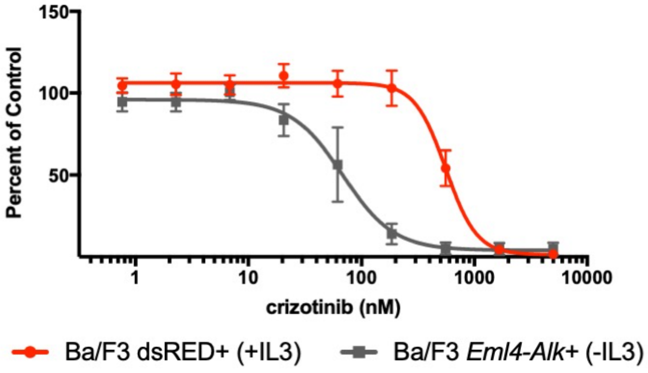

Cell Line	crizotinib IC50 (nM)
Ba/F3 dsRED+	557.5
Ba/F3 *Eml4-Alk*+	68.2

D

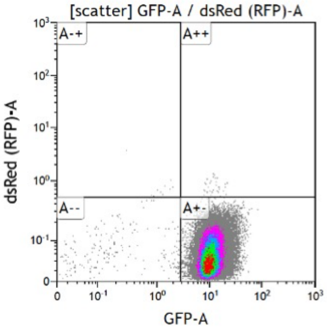

IL3-independent *Eml4-Alk*+ Ba/F3 cells

Supplementary Figure 2

Biology Open • Supplementary information

Fig. S2. Ba/F3 screening method selects and enriches for cells with CRISPR/Cas9 generated fusion oncogenes using validated *Eml4-Alk* sgRNAs. A. Flow cytometry for dsRED and GFP after transfection with *Eml4-Alk* sgRNAs **B.** Genomic PCR using fusion specific primers, or non-rearranged *Eml4* primers in Ba/F3 + *Eml4-Alk* sgRNAs, parental Ba/F3 or Ba/F3-dsRED$^{fl/fl}$-eGFP cells (indicated as Ba/F3 dsRED+). "*Eml4-Alk* positive control" = DNA derived from mouse tumors confirmed to have *Eml4-Alk* rearrangements. **C.** MTS proliferation assay in Ba/F3-dsRED$^{fl/fl}$-eGFP cells and Ba/F3 *Eml4-Alk*+ cells treated with increasing concentrations of crizotinib. **D.** Flow cytometry for dsRED and GFP after IL3 independence.

Biology Open • Supplementary information

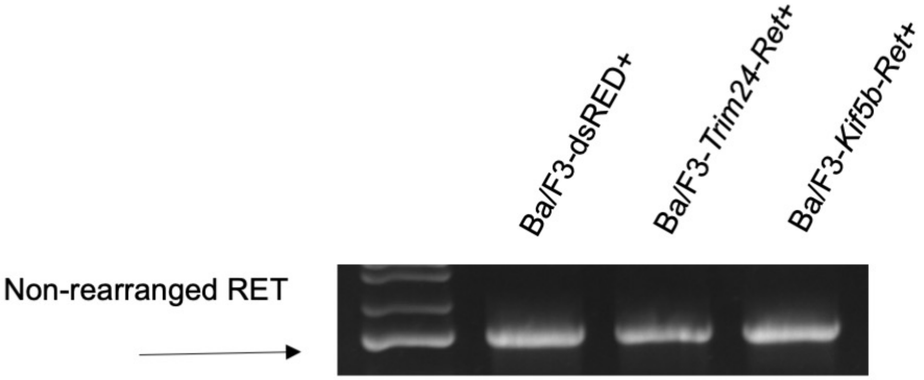

Supplementary Figure 3

Fig. S3. Ba/F3-dsRED$^{fl/fl}$-eGFP cells with *Kif5b-Ret* and *Trim24-Ret* rearrangements contain a copy of wild type, non-rearranged *Ret*. Genomic PCR of *Ret* across intended fusion breakpoint.

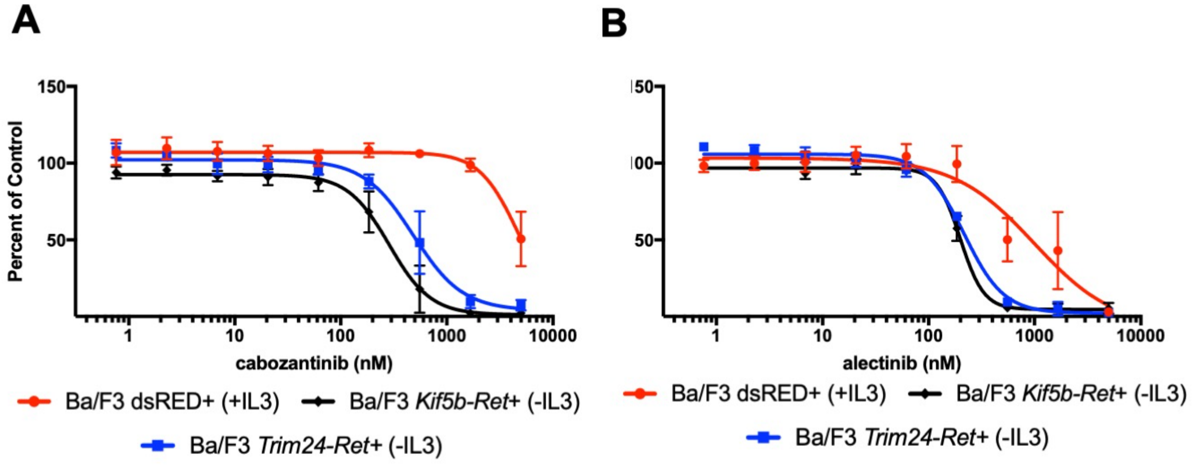

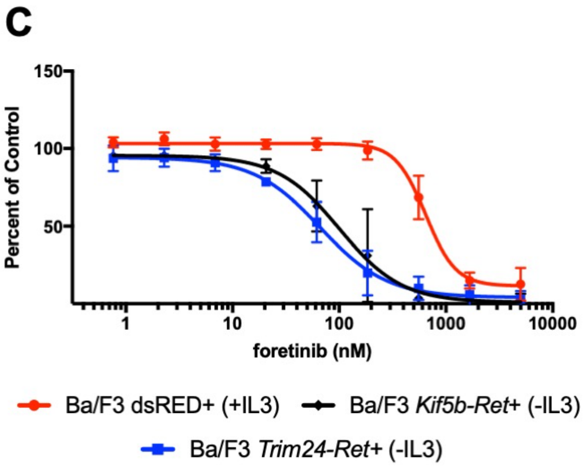

D

Cell Line	Inhibitor	IC50 (nM)
Ba/F3 dsRED+	cabozantinib	4648
Ba/F3 *Kif5b-Ret*+		287
Ba/F3 *Trim24-Ret*+		484
Ba/F3 dsRED+	alectinib	1000
Ba/F3 *Kif5b-Ret*+		199
Ba/F3 *Trim24-Ret*+		216
Ba/F3 dsRED+	foretinib	647
Ba/F3 *Kif5b-Ret*+		101
Ba/F3 *Trim24-Ret*+		66

Supplementary Figure 4

Fig. S4. Ba/F3-dsRED$^{fl/fl}$-eGFP cells with *Ret* rearrangements are sensitive to multiple RET inhibitors. A-C MTS proliferation assay in cells treated with increasing concentrations of (**A**) cabozantinib (**B**) alectinib or (**C**) foretinib. N=3 error bars represent ± SEM. **D.** Chart of IC_{50} values.

Table S1. Table of sgRNA sequences.

**sgRNA Sequences**		
Rearrangement	sgRNA 1 (5' partner)	sgRNA 2 (3' partner)
Kif5b-Ret	5'-GTTAAGTGAAAATCTTCAACG-3'	5'-GCTATACCCACATAAGCCCCA-3'
Trim24-Ret	5'-GATTGCTGAATAACCGCATAA-3'	5'-GTTACCCTTGGGAACGTTACA-3'
Tpm3-Ntrk1	5'-GTGCAAGTCTAGCATTAACAC-3'	5'-GCTAGCTGGGACCCCGAAGTG-3'

Biology Open • Supplementary information
